# Frontline pembrolizumab monotherapy for metastatic non-small cell lung cancer with PD-L1 expression ≥50%: real-world outcomes in a US community oncology setting

**DOI:** 10.3389/fonc.2024.1298603

**Published:** 2024-03-08

**Authors:** Shirish M. Gadgeel, Pragya Rai, Srinivas Annavarapu, Sartaj Alam, Jerome H. Goldschmidt, Howard (Jack) West, Melissa Santorelli, Renata Eiras Martins

**Affiliations:** ^1^ Division of Hematology/Oncology, Henry Ford Cancer Center Institute, Detroit, MI, United States; ^2^ Center for Observational and Real-world Evidence, Merck & Co., Inc., Rahway, NJ, United States; ^3^ Real-World Research Ontada, Boston, MA, United States, United States; ^4^ Medical Oncology/Hematology, Blue Ridge Cancer Centers/The US Oncology Network, Blacksburg, VA, United States; ^5^ Department of Medical Oncology and Therapeutics Research, City of Hope, Comprehensive Cancer Center, Duarte, CA, United States; ^6^ Clinical Research, Merck & Co., Inc., Rahway, NJ, United States

**Keywords:** non-small cell lung cancer, pembrolizumab monotherapy, overall survival, real-world time on treatment, reasons for discontinuation

## Abstract

**Background:**

This study investigated real-world time on treatment (rwToT) and overall survival (OS) for patients with metastatic non-small cell lung cancer (mNSCLC) who initiated first-line (1L) pembrolizumab monotherapy. We also explored discontinuation reasons and subsequent treatments, stratified by number of cycles among those who completed ≥17 cycles of 1L pembrolizumab.

**Methods:**

Patients with mNSCLC without actionable genetic aberrations, Eastern Cooperative Oncology Group performance status (ECOG PS) 0-2 and unknown, and PD-L1 TPS ≥ 50% starting 1L pembrolizumab monotherapy between 24-Oct-2016 and 31-Dec-2018 within The US Oncology Network were identified retrospectively and evaluated using structured data, with a data cutoff of 30-Sep-2021. Patient characteristics and disposition were summarized using descriptive statistics. OS and rwToT were evaluated using Kaplan-Meier method for all ECOG PS and PS 0-1. A subgroup of patients who completed ≥17 cycles were evaluated using supplemental chart review data to discern reasons for discontinuation.

**Results:**

Of the 505 patients with mNSCLC with PD-L1 TPS ≥50%, 61% had ECOG PS 0-1, 23% had ECOG PS 2, and 65% had nonsquamous histology. Median rwToT and OS of pembrolizumab were 7.0 (95% CI, 6.0–8.4) months and 24.5 (95% CI, 20.1–29.3) months, respectively. In the subgroup with ECOG PS 0-1, they were 7.6 months (95% CI, 6.2-9.2) and 28.8 months (95% CI, 22.4-37.5), respectively. Of the 103 patients who completed ≥17 cycles, 57 (55.3%) patients received 17 – 34 cycles and 46 (44.7%) patients received ≥35 cycles. Approximately 7.7% of the study population received pembrolizumab beyond 35 cycles. Most common reasons for discontinuation were disease progression (38.6%) and toxicity (19.3%) among patients who received 17-34 cycles of pembrolizumab, and disease progression (13.0%) and completion of therapy (10.9%) among patients who received ≥35 cycles.

**Conclusion:**

Consistent with findings from KEYNOTE-024 and other real-world studies, this study demonstrates the long-term effectiveness of pembrolizumab monotherapy as 1L treatment for mNSCLC with PD-L1 TPS ≥50%. Among patients who completed ≥17 cycles, nearly half completed ≥35 cycles. Disease progression and toxicity were the most common reasons for discontinuation among patients who received 17-34 cycles of pembrolizumab. Reasons for discontinuation beyond 35 cycles need further exploration.

## 1 Introduction

Lung cancer is the third most common type of cancer, representing 12.3% of new cancer cases in the United States (US), and is the deadliest cancer with an estimated 127,070 deaths in 2023 (1). Notably, approximately 50% of cases are diagnosed at advanced stages (unresectable stage IIIB/C and stage IV) (2). Historically, the standard of care in the front-line setting for metastatic non-small cell lung cancer (mNSCLC) consisted of platinum-based doublet chemotherapy regimens. However, these have been associated with considerable toxicity and low efficacy (1-year survival 30%-40%, overall survival [OS] 7.8-13 months) (3–5). This highlighted the need for novel therapies, which led to the development of immune checkpoint inhibitors targeting the programmed death receptor-1 (PD-1) or the programmed death ligand-1 (PD-L1).

In October 2015, pembrolizumab became the first immune checkpoint inhibitor approved by the US Food and Drug Administration (FDA) for mNSCLC that had progressed after other treatments and expressed PD-L1 (6). In October 2016, the US FDA expanded the indication to first-line (1L) treatment of patients with mNSCLC with PD-L1 tumor proportion score (TPS) ≥50% without actionable EGFR mutations or ALK rearrangements (7). The 1L approval was based on KEYNOTE-024, which compared pembrolizumab monotherapy with platinum-based chemotherapy as 1L treatment of patients with PD-L1 TPS ≥50% mNSCLC without EGFR sensitizing mutation or ALK translocation. Treatment was given until unacceptable levels of toxicity developed, until patients developed progressive disease per RECIST 1.1, or for up to 24 months (35 cycles, 200 mg every 3 weeks). Pembrolizumab was associated with a significantly longer OS (median OS was not reached in either cohort; hazard ratio=0.60; 95% confidence interval [CI], 0.41-0.89; survival at 6 months was 80.2% for pembrolizumab and 72.4% for chemotherapy) and fewer adverse events (AEs) than platinum-based chemotherapy (8). A 5-year follow-up OS analysis reported a median OS of 26.3 months (95% CI, 18.3-40.4) for pembrolizumab compared to 13.4 months (95% CI, 9.4-18.3) for chemotherapy (9).

In the KEYNOTE-024 5-year OS analysis, 25.8% (39/151) of patients completed 35 cycles of pembrolizumab (9). Based on the trial protocols and results, the recommended dosage section of the pembrolizumab US package insert specifies that pembrolizumab treatment should continue until disease progression, unacceptable toxicity, or up to 24 months (10). However, data are sparse on how the recommended 24-month treatment duration is implemented in clinical practice. Velcheti and colleagues investigated 1L pembrolizumab monotherapy patients with PD-L1 TPS ≥50% with Eastern Cooperative Oncology Group (ECOG) performance status (PS) 0-2 and no EGFR/ALK/ROS1 alterations from a longitudinal Flatiron Health electronic health records (EHR) database chart review (11). In the ECOG PS 0-1 and 2 cohorts respectively, 22.1% (95% CI, 19.1%-25.3%) and 9.9% (95% CI, 6.1%-14.6%) remained on pembrolizumab for 24 months. Reasons for treatment discontinuation among patients initiating pembrolizumab monotherapy were previously reviewed; Velcheti and colleagues found that most patients discontinued due to progression (46%), while the second most frequent reason was adverse effect of therapy (23%) (12). However, reasons for discontinuation were not evaluated by the number of cycles received.

To further understand treatment patterns in clinical practice in this patient population, we assessed real-world time on treatment (rwToT) and reasons for treatment discontinuation associated with 1L pembrolizumab monotherapy in patients with mNSCLC with high levels of PD-L1 expression. We were especially interested in understanding the extent to which patients stopped therapy after completing the recommended maximum course of therapy (up to 24 months) and reasons for treatment discontinuation for patients who completed at least 17 cycles and in patients who completed 35 cycles (up to 24 months). We also investigated baseline characteristics, subsequent treatments stratified by treatment duration and OS in the overall patient cohort.

## 2 Methods

### 2.1 Data source

This study was conducted using structured data from the EHR at The US Oncology Network and supplemented with chart review to capture enhanced clinical data. The US Oncology Network includes 1,400 affiliated physicians operating in over 500 sites of care, with approximately 1.2 million US patients with cancer treated annually (13). Data from structured fields in the EHR were collected via programmatic queries of The US Oncology Network’s iKnowMed (iKM) EHR database. Vital status was also captured from the Limited Access Death Master File to supplement available death dates from the structured data in the iKM EHR. Data validation consisted of, but was not limited to, quality control checks for appropriate values, logical sequences, and quantity of missing values. Missing data were identified and reported as percentages for all variables.

### 2.2 Study design and patients

**Figure 1:**
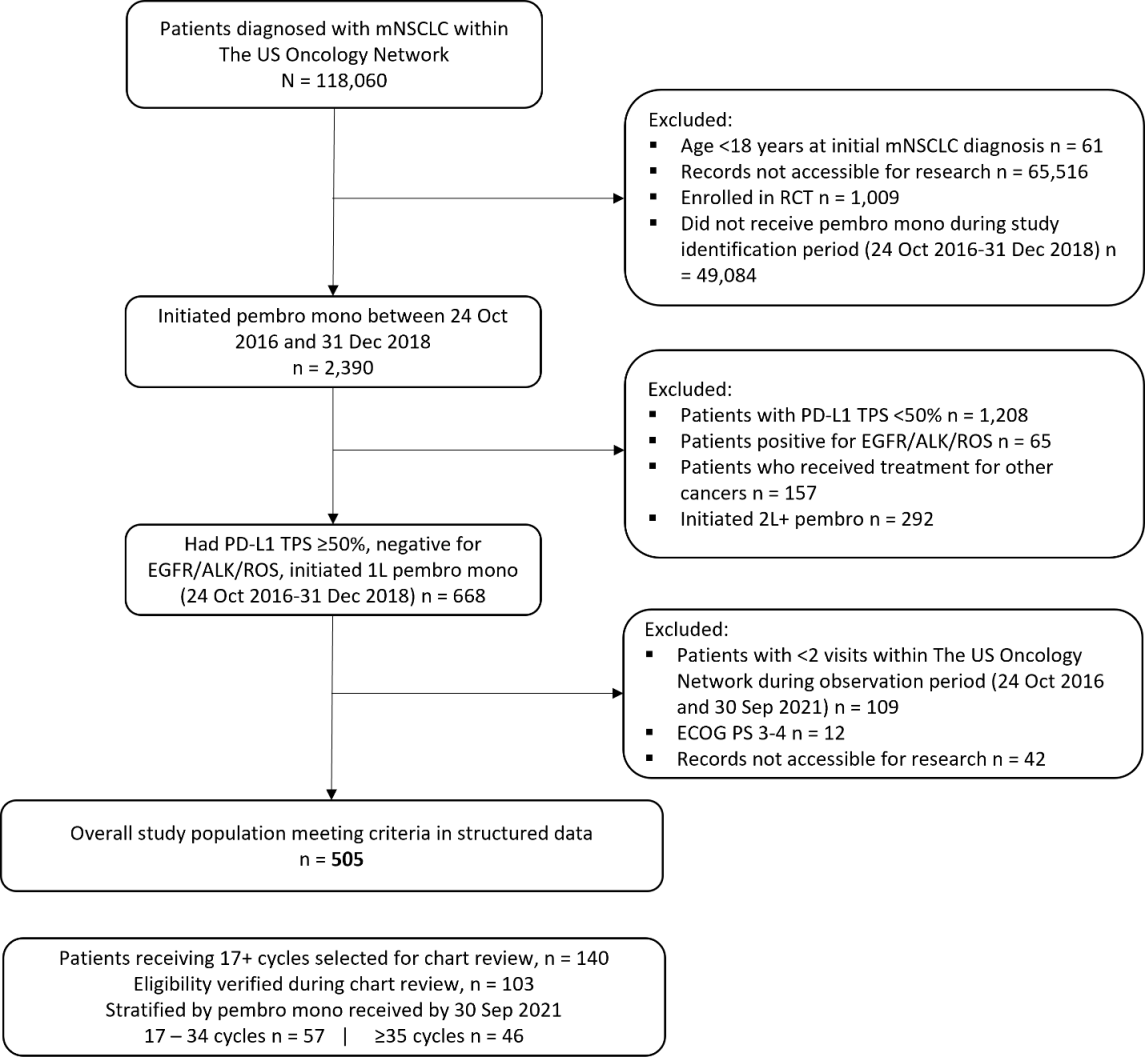
Attrition of overall study population. 1L, first-line treatment; 2L+, second-line and beyond treatment; ALK, anaplastic lymphoma kinase; ECOG PS, Eastern Cooperative Oncology Group performance status; EGFR, epidermal growth factor receptor; mNSCLC, metastatic non-small cell lung cancer; PD-L1, programmed death receptor ligand-1; RCT, randomized controlled trial; ROS1, ROS proto-oncogene 1; TPS, tumor proportion score.

This was a retrospective observational cohort study design. Adult patients (≥18 years) with a confirmed mNSCLC diagnosis and PD-L1 TPS≥50% within The US Oncology Network were identified (**Figure 1**). The index date was defined as treatment initiation with 1L pembrolizumab monotherapy between 24 October 2016 and 31 December 2018 (i.e., the study identification period). Patients were followed from index to data cutoff (30 September 2021), the date of last visit within The US Oncology Network, or date of death, whichever came first. Patients also had to have at least two visits after the initiation of 1L pembrolizumab within The US Oncology Network during the follow-up period. Patients with non-squamous histology were required to have a documented lack of EGFR/ALK aberrations while patients with other histology (squamous, not otherwise specified, other) were required to have either a lack of EGFR/ALK aberrations or unknown EGFR/ALK aberration status. Patients with any histology were required to have a documented lack of ROS1 gene alteration or unknown ROS1 status. Patients were excluded if they received other systemic treatments within the 28-day period following mNSCLC diagnosis, were enrolled in interventional clinical trials at any time during the study observation period, had received their pembrolizumab through a clinical trial, were receiving treatment for other documented primary cancer diagnoses prior to or during the study observation period, or had ECOG PS 3 or 4. The study variables were derived using structured fields in the database.

We operationalized years into number of cycles, which was calculated as number of doses of pembrolizumab received, with a maximum allowable gap of 120 days. If a patient did not receive pembrolizumab doses for 120 days, the treatment was considered discontinued, and any further administrations after 120 days were defined as a later line of therapy (LOT). Therefore, based on a cycle duration of 200 mg every 3 weeks (10), patients with 35 cycles would have received at least 2 years of pembrolizumab therapy. Patients with 17 cycles would have received at least 1 year of pembrolizumab therapy. A few patients (n<15) received a 400-mg dose every 6 weeks. For these patients, 1 year of pembrolizumab was defined as administration of 8 cycles and 2 years of pembrolizumab as administration of 17 cycles.

For analyses using structured data, LOT were assigned using an algorithm based on previously published literature (14). A line regimen window of 28 days was used. When a gap in drug episodes of more than 120 days occurred, the LOT number was advanced. Periods without drug administrations from 42-119 days were considered as gaps.

### 2.3 Analyses

Descriptive statistics such as frequency and percentage were used to describe the baseline characteristics, patient disposition, and reasons for discontinuation.

OS was defined as the interval between the index event and the date of death (any cause) as documented in the EHR and LADMF. Patients who did not die within the study observation period were censored on the study end date, or the last contact date available in the dataset, whichever occurred first.

rwTOT was defined as the length of time between the first and last administration date before discontinuation of pembrolizumab (last administration – first administration + 1 day). Patients who had a record of initiating a next LOT after a pembrolizumab-based regimen or had a date of death while receiving pembrolizumab were considered as having discontinued on the last pembrolizumab administration date. If none of these events were identified, then having a gap of >120 days between the last administration date of pembrolizumab and last known activity date was considered as having discontinued at the last pembrolizumab administration date. Patients with <120 days between the last administration date of pembrolizumab and the last known activity date were considered as having discontinued if there was a physician order to discontinue pembrolizumab. If none of the above discontinuation criteria were met, patients were considered censored at their last administration date.

OS and rwToT were evaluated using Kaplan-Meier methods based on previously published literature and were evaluated for the overall study population as well as for a subgroup with ECOG PS 0-1 (15). Landmark estimates were presented at 6-month intervals up to a maximum of 36 months. Statistical analysis was conducted using SAS 9.4 (SAS Institute, Cary NC). The Strengthening the Reporting of Observational Studies in Epidemiology (STROBE) guidelines were used for reporting results from this study (16).

This study, which used retrospective patient data, was reviewed and approved by US Oncology Institutional Review Board. Institutional Review Board and Compliance/Privacy approvals were obtained prior to initiation of this study. This study involved the analysis of existing data and records, which were analyzed such that research participants could not be directly identified.

## 3 Results

### 3.1 Results by overall cohort

A total of 505 patients were identified, after applying the inclusion and exclusion criteria (**Figure 1**).

#### 3.1.1 Baseline characteristics and patient disposition

Details of demographic and clinical characteristics are presented in **Table 1**. At the data cutoff (30 September 2021), the median (range) follow-up duration for the overall cohort from index was 12.8 (0.2, 57.5) months. Of the 505 patients in the overall cohort who were diagnosed with mNSCLC with PD-L1 TPS ≥50% and treated with 1L pembrolizumab monotherapy, the majority were older than 65 years old (73.3%), White (74.5%), and former smokers (61.6%). Approximately two-thirds received treatment at practices in the West and South regions. A lower proportion of practice locations in the Northeast region is an artifact of the lesser presence of The US Oncology Network in that region.

**Table 1:** Baseline demographic characteristics of patients with mNSCLC and PD-L1 expression ≥50% that initiated 1L pembrolizumab monotherapy, overall and by category of number of cycles.

Variable	Overall study population	17 to 34 cycles of pembrolizumab	≥35 cycles of pembrolizumab
**Total patient count**	505	57	46
Age at index, years			
Mean (SD)	71.2 (10.3)	72.1 (10.8)	69.3 (12.5)
Age group, n (%)			
<65 years	135 (26.7)	14 (24.6)	18 (39.1)
≥65 years	370 (73.3)	43 (75.4)	28 (60.9)
<75 years	306 (60.6)	37 (64.9)	28 (60.9)
≥75 years	199 (39.4)	20 (35.1)	18 (39.1)
Gender, n (%)			
Male	247 (48.9)	28 (49.1)	25 (54.3)
Female	258 (51.1)	29 (50.9)	21 (45.7)
Race, n (%)			
White	376 (74.5)	39 (68.4)	31 (67.4)
Black	45 (8.9)	5 (8.8)	11 (23.9)
Other/ Not documented	84 (16.7)	13 (22.8)	4 (8.7)
BMI category, n (%)			
Underweight (BMI<18.5 kg/m^2^)	27 (5.3)	2 (3.5)	3 (6.5)
Normal (BMI 18.5-24.9 kg/m^2^)	206 (40.8)	23 (40.4)	14 (30.4)
Overweight (BMI 25-29.9 kg/m^2^)	148 (29.3)	9 (15.8)	20 (43.5)
Obese (BMI≥30 kg/m^2^)	105 (20.8)	17 (29.8)	8 (17.4)
Not documented (missing height or weight data)	19 (3.8)	6 (10.5)	1 (2.2)
Smoking history, n (%)			
Current smoker	116 (23.0)	13 (22.8)	9 (19.6)
Former smoker	311 (61.6)	32 (56.1)	25 (54.3)
Never smoker	47 (9.3)	5 (8.8)	5 (10.9)
Not documented	31 (6.1)	7 (12.3)	7 (15.2)
Practice location, n (%)			
Midwest	148 (29.3)	15 (26.3)	20 (43.5)
Northeast	34 (6.7)	4 (7.0)	3 (6.5)
South	152 (30.1)	18 (31.6)	13 (28.3)
West	171 (33.9)	20 (35.1)	10 (21.7)
Stage at initial NSCLC diagnosis, n (%)			
I	38 (7.5)	6 (10.5)	2 (4.3)
II	21 (4.2)	1 (1.8)	2 (4.3)
III	28 (5.6)	1 (1.8)	3 (6.5)
IV	390 (77.2)	48 (84.2)	37 (80.4)
Not documented	28 (5.6)	1 (1.8)	2 (4.3)
Presence of brain metastasis, n (%)	64 (12.7)	8 (14.0)	10 (21.7)
ECOG PS score, n (%)			
0-1	309 (61.2)	39 (68.4)	22 (47.8)
2	114 (22.6)	11 (19.3)	14 (30.4)
Not documented	82 (16.2)	7 (12.3)	10 (21.7)
Histology, n (%)			
Squamous	94 (18.6)	11 (19.3)	5 (10.9)
Non-squamous	329 (65.1)	38 (66.7)	33 (71.7)
Other	8 (1.6)	8 (14.0)	8 (17.4)
Not documented	74 (14.7)	0 (0)	0 (0)
Charlson Comorbidity Index			
Mean (SD)	1.2 (1.3)	1.2 (1.2)	1.1 (1.4)

BMI, body mass index; ECOG PS, Eastern Cooperative Oncology Group performance status; NSCLC, non-small cell lung cancer; SD, standard deviation.

A substantial proportion of patients had ECOG PS 0-1 (61.2%) and nonsquamous histology (65.1%).

In the overall cohort (n=505), 161 (31.9%) patients died before initiating subsequent treatment. Among the 344 remaining patients, 161 (46.8%) initiated subsequent treatment. The most frequently used subsequent regimens were pemetrexed + platinum (33%), paclitaxel + platinum (14%) and pembrolizumab monotherapy (14%) (**Supplementary Table 1**). The most frequent categories of subsequent treatments were chemotherapy (60.9%), immuno-oncology therapy (IO) + chemotherapy (14.9%), and IO monotherapy (14.3%). Among patients on subsequent treatments, 86 (53.4%) deaths were reported (**Supplementary Table 1**).

#### 3.1.2 Overall survival

**Figure 2:**
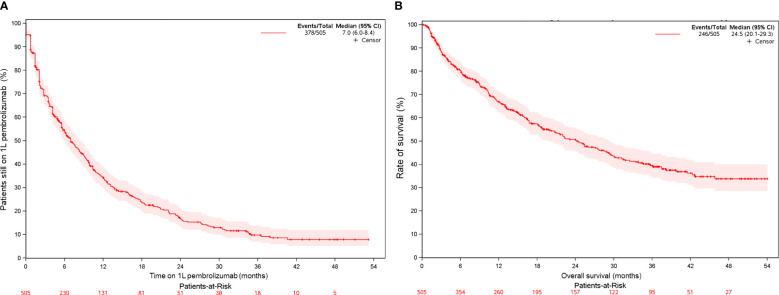
Kaplan-Meier curve for patients with mNSCLC and PD-L1 TPS ≥50% (overall study population) of **(A)** time on 1L pembrolizumab monotherapy **(B)** overall survival on 1L pembrolizumab monotherapy.

The median OS with 1L pembrolizumab was 24.5 months (95% CI, 20.1-29.3) (**Table 2**, **Figure 2B**). Survival rates at 12 months and 24 months were 66.8% (95% CI, 62.5%-71.4%) and 50.6% (95% CI, 45.9%-55.9%), respectively. In line with KEYNOTE-024, we also examined OS with 1L pembrolizumab among patients with ECOG PS 0-1 (n=309) and observed a median OS of 28.8 months (95% CI, 22.4-37.5), with survival probability at 12 and 24 months as 73.2% (95% CI, 68.1%-78.7%) and 55.8% (95% CI, 49.8%-62.5%), respectively. (**Supplementary Table 2**, **Supplementary Figure 1A**).

**Table 2:** Time on treatment and overall survival among overall study population of patients with mNSCLC and PD-L1 expression ≥50% (n=505) that initiated 1L pembrolizumab monotherapy (Kaplan-Meier estimate).

Time on treatment variables	N = 505
Discontinuations	378
**Median rwToT (95% CI), months**	7.0 (6.0-8.4)
**On-treatment rates, % (95% CI)**	
6 months	54.6 (50.2-59.3)
12 months	34.2 (30.0-39.1)
18 months	23.7 (19.9-28.3)
24 months	16.6 (13.2-20.9)
30 months	13.0 (9.9-17.0)
36 months	9.8 (6.9-13.7)
Overall survival variables	N = 505
Deaths	246
**Median OS (95% CI), months**	24.5 (20.1-29.3)
**Survival rates, % (95% CI)**	
6 months	80.5 (76.9-84.1)
12 months	66.8 (62.5-71.4)
18 months	57.5 (52.9-62.5)
24 months	50.6 (45.9-55.9)
30 months	43.5 (38.7-49.0)
36 months	39.4 (34.5-45.0)

CI, confidence interval; OS, overall survival; rwToT, real-world time on treatment.

#### 3.1.3 rwToT with pembrolizumab monotherapy

The median rwToT with 1L pembrolizumab was 7.0 months (95% CI, 6.0-8.4) (**Table 2**, **Figure 2A**). On-treatment rates at 12 months and 24 months were 34.2% (95% CI, 30.0%–39.1%) and 16.6% (95% CI, 13.2%–20.9%), respectively. In line with KEYNOTE-024, we also examined rwToT with 1L pembrolizumab among patients with ECOG PS 0-1 (n = 309) and observed a median of 7.6 months (95% CI, 6.2-9.2), with on-treatment probability at 12 and 24 months as 34.5% (95% CI, 29.2%-40.8%) and 15.0% (95% CI, 10.9%-20.6%), respectively (**Supplementary Table 2**, **Supplementary Figure 1B**).

To further understand the number of patients treated by cycles, we analyzed the data by number of cycles, as presented in subsequent sections.

### 3.2 Results by number of cycles (17-34 cycles and ≥35 cycles)/treatment duration

Of the 505 patients, a total of 103 patients who received at least 17 cycles (or 1 year) of 1L pembrolizumab and eligible for chart review were identified, of whom 57 patients received 17 to 34 cycles and 46 patients received ≥35 cycles (or ≥2 years; 5 patients received 35 cycles and 41 patients received ≥36 cycles) of pembrolizumab monotherapy. Approximately 7.7% of the overall study population received pembrolizumab monotherapy beyond 35 cycles. The median numbers of 1L pembrolizumab cycles were 24.0 and 46.0 for the 17-34 and ≥35 cycle cohorts respectively.

#### 3.2.1 Baseline characteristics stratified by treatment duration

The baseline characteristics for patients across the two duration-of-treatment groups were consistent with the overall population, except age and ECOG PS (**Table 1**). Patients in the 17-34 cycle group were slightly numerically older than the ≥35 cycle group (mean ± standard deviation [SD]: 72.1 ± 10.8 years vs 69.3 ± 12.5 years respectively) and a numerically lower proportion presented with ECOG PS 2 (19.3% vs 30.4% respectively). Details on all baseline characteristics are shown in **Table 1**.

#### 3.2.2 Patient disposition stratified by treatment duration

Among the patients who received 17-34 cycles, approximately 30% initiated a next LOT (**Table 3**). Among those patients, carboplatin-based combinations of chemotherapy comprised the most common regimen class. The minority received IO-based subsequent treatment. Nearly all patients receiving 35 cycles or more of 1L pembrolizumab monotherapy did not initiate a next LOT (89.1%), with 47.8% continuing treatment at the end of the follow-up period.

**Table 3:** Patient disposition and treatment patterns by category of number of 1L pembrolizumab monotherapy cycles received.

	17-34 Cycles n=57	≥35 Cycles n=46
**Did not initiate next line of therapy, n (%)**	40 (70.2)	41 (89.1)
Ongoing treatment	–	22
No ongoing treatment	40	19
No progression	19	7
Progression	21	12
Deaths	13	5
**Initiated next line of therapy, n (%)**	17 (29.8)	5 (10.9)
*** Chemotherapy, n (%)***	***11 (19.3)***	***3 (6.5)***
Carboplatin + Pemetrexed	5 (8.8)	2 (4.3)
Carboplatin + Paclitaxel	3 (5.3)	0
Carboplatin + Gemcitabine	1 (1.8)	0
Pemetrexed	2 (3.5)	0
Docetaxel + Ramucirumab	0	1 (2.2)
*** IO + chemotherapy, n (%)***	***3 (5.3)***	***2 (4.3)***
Carboplatin + Pembrolizumab + Pemetrexed	3 (5.3)	1 (2.2)
Bevacizumab + Pembrolizumab	0	1 (2.2)
*** IO monotherapy, n (%)***	***3 (5.3)***	***0***
Atezolizumab	1 (1.8)	0
Nivolumab	1 (1.8)	0
Pembrolizumab	1 (1.8)	0

IO, immuno-oncology therapy.

#### 3.2.3 Reasons for discontinuation stratified by treatment duration

Among the 17-34 cycle cohort, the three most common reasons for discontinuation were provider-documented disease progression (38.6%), toxicity (19.3%) and decline in performance status (12.3%) (**Table 4**). While provider-documented disease progression (13.0%), completion of therapy (10.9%) and decline in performance status (8.7%) were the three most common reasons for discontinuation among the ≥35-cycle cohort, sample sizes were low for accurate determination of reasons.

**Table 4:** Reasons for discontinuation of 1L pembrolizumab among patients with mNSCLC and PD-L1 expression ≥50%.

Variables	17-34 Cycles N=57	≥35 Cycles N=46
Pembrolizumab number of cycles		
** Mean (SD)**	24.8 (5.3)	49.8 (12.8)
** Median (range)**	24.0 (17.0, 34.0)	46.0 (35.0, 88.0)
* Treatment Ongoing, n*	*0*	*22*
Patients who discontinued, n (%)	57 (100)	24 (52.2)
Reasons for discontinuation, n (%)		
Provider-documented disease progression	22 (38.6)	6 (13.0)
Toxicity	11 (19.3)	1 (2.2)
Loss to follow-up	8 (14.0)	2 (4.3)
Decline in performance status	7 (12.3)	4 (8.7)
Completion of therapy	5 (8.8)	5 (10.9)
Death	3 (5.3)	4 (8.7)
Physician preference	0	2 (4.3)
Patient preference	2 (3.5)	0
Complete response	1 (1.8)	0
Not documented	12 (21.1)	4 (8.7)

SD, standard deviation.

## 4 Discussion

This study highlights the long-term effectiveness of frontline pembrolizumab monotherapy in patients with mNSCLC and PD-L1 TPS ≥50%. The median rwToT of 7 months and OS of 24.5 months associated with 1L pembrolizumab monotherapy in our study are consistent with KEYNOTE-024 and other real-world studies, for a ECOG PS 0-1 subgroup as well as for the total study population with ECOG PS 0-2. The 505 patients in our study population had a minimum potential follow up period of 3 years; 49% (n=246) had a record of death during this time. In KEYNOTE-024, the 3-year estimate of survival was 44% (9). Velcheti and colleagues investigated 1L pembrolizumab monotherapy patients with PD-L1 TPS ≥50% with ECOG PS 0-1 and no EGFR/ALK/ROS1 alterations from a longitudinal Flatiron Health EHR database chart review (17). The study reported a 12-month survival rate of approximately 64% (17) with a median follow-up of 15.5 months, while the 12-month survival rate in our study was 67% with a follow-up of 12.8 months. Velcheti and colleagues then analyzed rwToT using the same database but with a median follow-up of 34 months (11). They found that patients with PD-L1 TPS ≥50% with no EGFR/ALK/ROS1 alterations had a median rwToT of 7.4 months (95% CI, 6.3-8.1) for those with ECOG 0-1, and the on-treatment rate at 24 months was 22.1% (95% CI, 19.1%-25.3%) (11). In our study, the median rwToT for those with ECOG PS 0-1 was 7.6 months (95% CI, 6.2-9.3) and the on-treatment rates at 12 and 24 months were 34.2% (30.0%-39.1%) and 15.0% (95% CI, 10.9%-20.6%). It is notable that most of the patients who discontinued did so in the first 12 months. Sun et al. performed a retrospective cohort study of the Flatiron database of patients who received frontline IO with or without chemotherapy for advanced/metastatic NSCLC. Among 14,406 patients who had initiated frontline IO, 2,765 patients were still on that treatment by 12-14 months and 1,091 by 2 years (18).

In our real-world study, the most frequent categories of subsequent treatments in the overall cohort were single agent or combination chemotherapy, IO + chemotherapy, and IO monotherapy. In KEYNOTE-024, the most common subsequent systemic therapy classes among those receiving 1L pembrolizumab were platinum-based chemotherapy combinations, single agent chemotherapy with or without antiangiogenic combinations and IO monotherapy or combination therapy (9) Velcheti and colleagues found that the most common systemic therapy classes in 2L were platinum-based chemotherapy and anti-PD-1/PD-L1 combination therapies (11).

Both our study population and KEYNOTE-024 investigated patients with mNSCLC with PD-L1 TPS ≥ 50% and negative for sensitizing ALK or EGFR aberrations. Both populations included patients with brain metastases (12.7% and 11.7% in our study and KEYNOTE-024 (9), respectively). The highest proportion of patients in each population were former smokers. At the same time, our study population also had some differences from that of the KEYNOTE-024 study: slightly older at index (median age 72 years vs 64.5 years in KEYNOTE-024) and included a higher proportion of female patients (51.1% vs 40.3% KEYNOTE-024). Also, KEYNOTE-024 included patients with an ECOG PS of 0 or 1, while 22.6% of our study population presented with an ECOG PS of 2. These demographics found in our study are consistent with other real-world studies utilizing other data sources (17, 19).

We further identified 103 patients who received ≥17 cycles of 1L pembrolizumab monotherapy. Among these patients, 45% received ≥35 cycles. Approximately 7.7% of patients received pembrolizumab beyond 35 cycles. While the common reasons for discontinuation among patients who received 17–34 cycles were discernable as disease progression and toxicity, the reasons could not be clearly elucidated for patients who received 35 cycles and beyond, in part due to the smaller number of patients in this group. Among the 154 patients receiving 1L pembrolizumab in KEYNOTE-024, 39 patients received 35 cycles, 34 completed treatment as per provider assessment, and 120 discontinued pembrolizumab; 77 (50%) patients received another course of systemic therapy, including 12 that received another course of pembrolizumab. In our study, among patients receiving ≥17 cycles of 1L pembrolizumab, 21.4% (n=22/103) received subsequent systemic therapy; among patients who received 17-34 cycles, the most common subsequent therapy class was chemotherapy (19%, n=11) followed by IO combination with chemotherapy and IO monotherapy (approximately 5% each, n=3 each). Selection of chemotherapy over IO-based therapies suggests that patients may have developed progressive disease on 1L pembrolizumab or have experienced toxicity such that rechallenge with IO would not have been feasible. However, conclusions about patterns of subsequent therapies in our study among patients who received ≥17 cycles of 1L pembrolizumab monotherapy are limited by small sample size, especially among those who received 35 cycles and beyond. As in our study, the leading reasons for discontinuation of 1L pembrolizumab in KEYNOTE-024 were progression and AE (46.8% and 20.1%, respectively). In the EHR database chart review study of Velcheti and colleagues, the leading reasons for discontinuation were disease progression, adverse effects of therapy, and disease-related symptoms. However, that study did not examine the reasons within the timeframe of the number of cycles completed (17).

Our results appeared to show disposition along the lines of real-world studies, although conclusions are limited by our study sample size and because a direct comparison between studies could not be performed. In the study by Velcheti and colleagues, 28% received subsequent systemic therapy, and the most common subsequent regimen class comprised anti-PD-1/PD-L1-based therapies. In our study, in the overall cohort, 32% of patients initiated a subsequent regimen, with 61% of these patients initiating single agent or combination chemotherapy. Among patients receiving ≥17 cycles of pembrolizumab, 21.4% received subsequent therapy; among patients who received 17-34 cycles, the most common class was chemotherapy. However, conclusions about patterns of subsequent therapies after 1L in our study are limited by small sample size. Velcheti and colleagues also found that approximately 10% of the overall population of patients with at least 2 years of follow-up received ≥35 cycles; among those with ECOG PS 0-1, 16% did (11). In our study, 10.5% of the overall study population had received ≥35 cycles. Velcheti and colleagues found that the median numbers of pembrolizumab cycles administered in the ECOG PS 0-1 and 2 cohorts were 10 and 3.5 among patients that had at least 2 years of follow-up (defined as duration of follow-up from 1L initiation to database cutoff) (11). In our study, the median numbers of 1L pembrolizumab cycles were 24.0 and 46.0 for the 17-34 and ≥35 cycle cohorts, respectively, and the median follow-up periods from index treatment were 25.2 months and 38.1 months, respectively.

### 4.1 Biases or limitations

The study has several limitations that must be noted, which are inherent to the real-world nature of the data and retrospective collection. First, sample sizes in subgroups within our study population were small. This precluded the ability to compare some of the treatment patterns and reasons for discontinuation when comparing groups by number of cycles received. Second, the iKM EHR is used for clinical practice purposes, and not solely for research purposes. Therefore, associations but not causality can be observed, and some study variables may not have been as completely documented. Third, while iKM data contain rich, detailed information about cancer patient journeys, patients enrolled in this study may have received medical services outside The US Oncology Network, which therefore may not have been captured in the study data. Fourth, while the study vital status data came from iKM and LADMF, which have been shown to have high concordance (20), these sources may not necessarily be comprehensive. Fifth, data entry errors at the point of care cannot be detected or corrected during analysis, which could lead to misclassification of diagnoses, events or procedures. Sixth, grades of AEs are not recorded in the iKM EHR, limiting comparison to other studies with AE grade data. Finally, community practices within The US Oncology Network may not necessarily reflect patterns of care within other community practices or academic settings in the US or other geographies.

### 4.2 Conclusion

Consistent with findings from KEYNOTE-024 and other real-world studies, this study reiterates the long-term effectiveness of pembrolizumab monotherapy for mNSCLC with PD-L1 TPS ≥50%. Disease progression and toxicity were the most common reasons for discontinuation among patients who received 17-34 cycles of pembrolizumab. Reasons for discontinuation beyond 35 cycles need further exploration.

## Data availability statement

The health data used to support the findings of this study are restricted by The US Oncology Research Institutional Review Board in order to protect patient privacy. For this reason, data used to support the findings of this study have not been made available.

## Ethics statement

Institutional Review Board and Compliance/Privacy approval was gained prior to initiation of the retrospective research. Since this project involved the analysis of existing data and records, study information was analyzed in such a manner that research participants could not be directly identified. Patient informed consent was not required due to the nature of the study design. Thus, exemption status and a waiver of informed consent were approved by The US Oncology, Inc. Institutional Review Board. Data were handled in compliance with HIPAA and the Health Information Technology for Economic and Clinical Health (HITECH) Act.

## Author contributions

SG: Investigation, Methodology, Validation, Writing – review & editing. PR: Conceptualization, Funding acquisition, Methodology, Project administration, Writing – original draft, Writing – review & editing. SAn: Data curation, Formal analysis, Methodology, Project administration, Validation, Writing – original draft, Writing – review & editing. SAl: Data curation, Formal analysis, Methodology, Writing – review & editing. JG: Investigation, Methodology, Validation, Writing – review & editing. HW: Investigation, Methodology, Validation, Writing – review & editing. MS: Conceptualization, Funding acquisition, Methodology, Writing – review & editing. RM: Investigation, Methodology, Validation, Writing – review & editing.
