# Supplementary material for: Frontline pembrolizumab monotherapy for metastatic non-small cell lung cancer with PD-L1 expression ≥50%: real-world outcomes in a US community oncology setting
Source: Front Oncol. 2024 Mar 8;14:1298603. doi: 10.3389/fonc.2024.1298603 (PMC10958653; doi:10.3389/fonc.2024.1298603)

**Supplemental Tables and Figures**

Supplementary Table 1. Disposition and subsequent regimens among patients with mNSCLC initiating pembrolizumab monotherapy

| **Variable** | **Overall study population** |
| --- | --- |
| **Total patient count** | 505 |
| **Disposition, n (% of all patients)** |  |
| Did not initiate next line of therapy | 344 (68.1) |
| Patients who did not initiate next line of therapy and died | 161 (31.9) |
| Initiated next line of therapy | 161 (31.9) |
| Patients who initiated next line of therapy and died | 86 (17.0) |
| **Distribution of subsequent regimens, n (% patients who initiated a subsequent regimen)** |  |
| ***Chemotherapy*** | 98 (60.9) |
| Pemetrexed+Platinum | 53 (32.9) |
| Paclitaxel+Platinum | 22 (13.7) |
| Gemcitabine+Platinum | 6 (3.7) |
| Other | 17 (10.6) |
| ***IO + chemotherapy*** | 24 (14.9) |
| Pembrolizumab+Pemetrexed+Platinum | 19 (11.8) |
| Other | 5 (3.1) |
| ***IO monotherapy*** | 23 (14.3) |
| Pembrolizumab | 22 (13.7) |
| Other | 1 (0.6) |
| ***Other*** | 16 (9.9) |

IO, immuno-oncology therapy

Supplementary Table 2. Kaplan-Meier estimate of time on 1L pembrolizumab monotherapy and overall survival among patients with mNSCLC and PD-L1 expression ≥50% and ECOG PS 0-1

| **Time on treatment variables** | **N = 309** |
| --- | --- |
| **Discontinuation, n** | 234 |
| **Median rwToT (95% CI), months** | 7.6 (6.2 – 9.3) |
| **On-treatment rates, % (95% CI)** |  |
| 6 months | 58.2 (52.7-64.2) |
| 12 months | 34.5 (29.2-40.8) |
| 18 months | 24.7 (19.9-30.8) |
| 24 months | 15.0 (10.9-20.6) |
| 30 months | 10.0 (6.6-15.1) |
| **Overall survival variables** | **N = 309** |
| **Deaths, n** | 139 |
| **Median OS (95% CI), months** | 28.8 (22.4-37.5) |
| **Survival rates (95% CI)** |  |
| 6 months | 85.1 (81.1-89.3) |
| 12 months | 73.2 (68.1-78.7) |
| 18 months | 61.3 (55.6-67.7) |
| 24 months | 55.8 (49.8-62.5) |
| 30 months | 47.9 (41.7-55.0) |

CI, confidence interval; ECOG PS, Eastern Cooperative Oncology Group performance status; OS, overall survival; rwToT, real-world time on treatment

Supplementary Figure 1. Kaplan-Meier curve for patients with mNSCLC and PD-L1 expression ≥50% and ECOG PS 0-1 (n=309): (a) Overall survival on 1L pembrolizumab monotherapy (b) Time on 1L pembrolizumab monotherapy

(a)

(b)
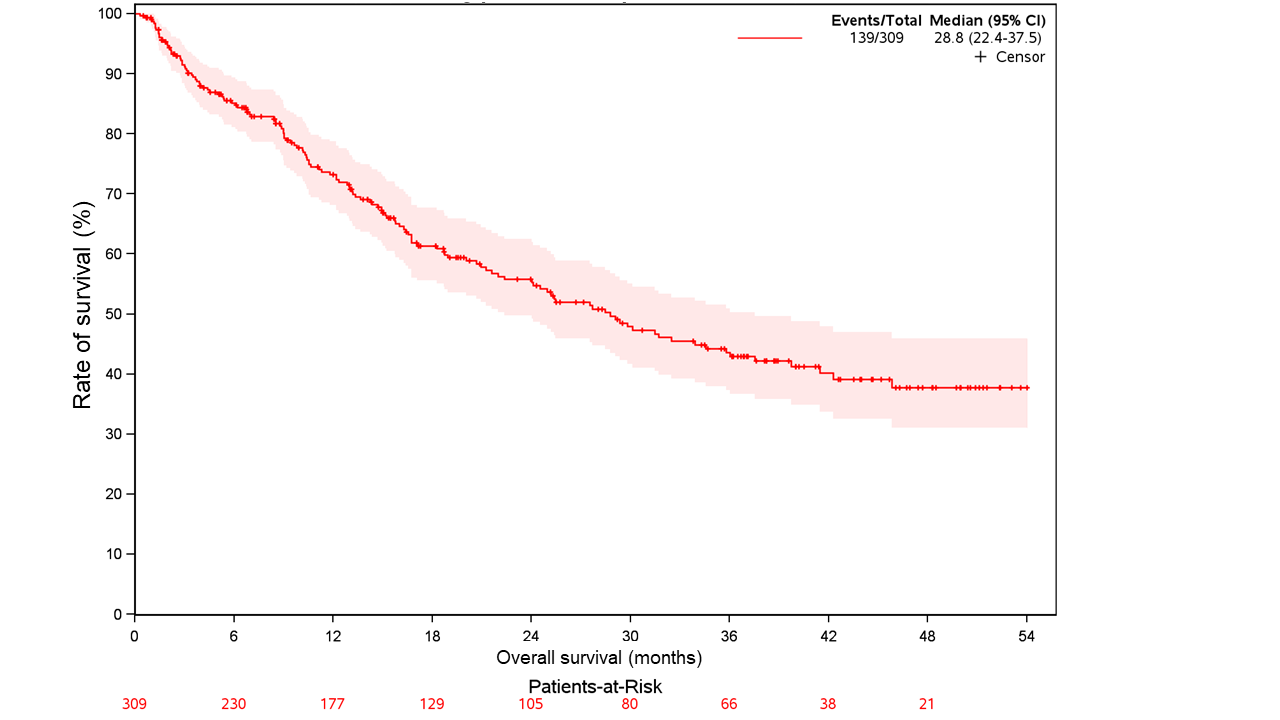


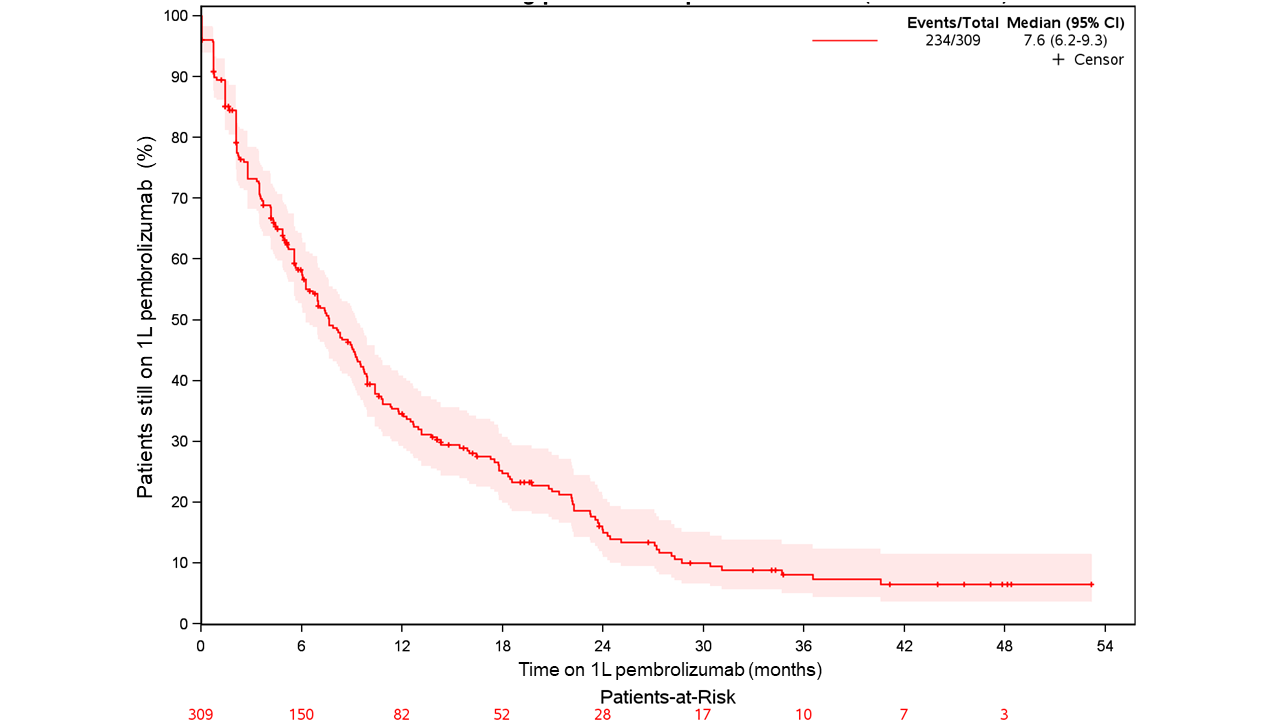

Supplement: Supplementary file 1 [file DataSheet_1.docx]
